# Determinants of National Health Insurance enrolment in Ghana across the life course: Are the results consistent between surveys?

**DOI:** 10.1186/s12939-018-0760-x

**Published:** 2018-04-23

**Authors:** Nele van der Wielen, Jane Falkingham, Andrew Amos Channon

**Affiliations:** 1 Centre for Research on Ageing, University of Southampton, Southampton, SO17 1BJ UK; 2 Social Statistics & Demography, ESRC Centre for Population Change, University of Southampton, Southampton, SO17 1BJ UK; 3 Social Statistics and Demography, University of Southampton, Southampton, SO17 1BJ UK

**Keywords:** Health Insurance, Universal Health Coverage, Ageing, Ghana

## Abstract

**Background:**

Ghana is currently undergoing a profound demographic transition, with large increases in the number of older adults in the population. Older adults require greater levels of healthcare as illness and disability increase with age. Ghana therefore provides an important and timely case study of policy implementation aimed at improving equal access to healthcare in the context of population ageing.

This paper examines the determinants of National Health Insurance (NHIS) enrolment in Ghana, using two different surveys and distinguishing between younger and older adults. Two surveys are used in order to investigate consistency in insurance enrolment. The comparison between age groups is aimed at understanding whether determinants differ for older adults. Previous studies have mainly focused on the enrolment of young and middle aged adults; thus by widening the focus to include older adults and taking into account differences in their demographic and socio-economic characteristics this paper provides a unique contribution to the literature.

**Methods:**

Using data from the 2007-2008 Study on Global Ageing and Adult Health (SAGE) and the 2012-2013 Ghanaian Living Standards Survey (GLSS) the determinants of NHIS enrolment among younger adults (aged 18-49) and older adults (aged 50 and over) are compared. Logistic regression explores the socio-economic and demographic determinants of NHIS enrolment and multinomial logistic regression investigates the correlates of insurance drop out.

**Results:**

Similar results for people aged 18-49 and people aged 50 plus were revealed, with older adults having a slightly lower probability of dropping out of insurance coverage compared to younger adults. Both surveys confirm that education and wealth increase the likelihood of NHIS affiliation. Further, residential differences in insurance coverage are found, with greater NHIS coverage in urban areas. The findings give assurance that both datasets (SAGE and GLSS) are suitable for research on insurance affiliation in Ghana.

**Conclusion:**

The paper indicates that although the gap in coverage among rich and poor and urban and rural residents appears to have decreased, these factors still determine NHIS coverage of younger and older adults. The same holds for education. Increasing efforts are needed to ensure equal access to healthcare.

## Background

Universal Health Coverage (UHC) aims to provide quality healthcare to all without financial hardship. Efforts to meet the aim of UHC will be greatly influenced by the global population ageing phenomenon. Older adults require greater levels of healthcare due to increasing illness and disability as ageing occurs, which complicates progress to UHC. Moreover, older adults in low and middle-income countries are often left without any regular income due to a lack of social pensions [1]. Social protection schemes in low and middle income countries are vital to the protection of older adults because in countries without social security systems, it is left to the individual to finance healthcare expenditures.

Ghana presents a crucial case study for the implementation of policies aimed at UHC in the context of population ageing in sub-Saharan Africa. Ghana is currently undergoing a profound demographic transition, with a large increase in the number of older adults. The percentage of people aged 60 and over is estimated to grow from 5% in 2015 to nearly 10% in 2050 [2]. In 2005 the country implemented a National Health Insurance Scheme (NHIS), designed to help improve equality of access to healthcare by offering affordable healthcare to all.

Prior to 2005, the Ghanaian health system was organised as a ‘cash and carry system’ with healthcare only obtained after payment. This was seen as highly inequitable and acted as a barrier to individuals getting timely and necessary treatment [3]. In response, the NHIS was set up which operates under a vision of being "a model of a sustainable, progressive and equitable social health insurance scheme in Africa and beyond" [4] and officially covers 95% of the common disease burden in its benefit package. Formally, it is a mandatory system; however, no penalties apply for not being a member of any health insurance scheme in Ghana. Those working in the formal labour sector subsidise the NHIS through an automatic payroll deduction [3, 5].

Previous research has been critical of the lack of evidence as to what influences uptake of the NHIS [6]. So far studies on NHIS enrolment have mainly focused on young and middle-aged adults, but have neglected to consider the extent to which these results can be generalised to older adults. For example, each of Blanchet, et al. [3], Ayitey, et al. [7] and Osei-Akoto and Adamba [5] examined demographic and socio economic factors that influence NHIS membership among adults without differentiating older adults from corresponding younger generations. This paper argues that it is important to consider younger and older adults separately due to differences in demographic and socio-economic characteristics as well as differences in health and disability status.

It is expected that older adults will make greater use of the NHIS than younger age groups as older adults are more vulnerable to disabilities and diseases. The 2010 Ghanaian Census showed that over 9% of older adults suffer from at least one disability compared to less than 3% of adults aged 18-49 years. Communicable diseases such as HIV and malaria are the main cause of disability-adjusted life-years (DALYs) until the age of 50, while for people aged 50 plus non-communicable diseases are the main cause of DALYs [8]. Furthermore, compared with younger adults, the educational status among older adults in Ghana is relatively low [9]. These differences in user characteristics can influence the uptake of health insurance significantly, which is why it is important to explore whether the correlates of NHIS enrolment differ between younger and older adults.

There is a lack of data focusing on older adults in sub-Saharan Africa which can explain the scarcity of studies looking at older adults in the region. Ghana has the advantage of having two nationally representative surveys that include a question on insurance coverage and sample all adults (inducing those aged 50 and over). They are the World Bank funded Ghanaian Living Standards Survey (GLSS) and the World Health Organization (WHO) Study on Global Ageing and Adult Health (SAGE). However, there are concerns about the reliability of different datasets for measuring NHIS enrolment, bringing into question the possibility of measuring health insurance coverage in Ghana accurately. Apoya and Marriott [10] estimated a total NHIS coverage of only 18% in 2009, while the National Health Insurance Authority (NHIA) reported a coverage of 62% in the same year [11]. The GLSS (2012/2013) estimated a coverage of 51% among people aged 18 and over and the SAGE (2007/2008) reported that 28% of survey participants (aged 18 and over) had NHIS coverage.

The objective of this paper is to investigate the correlates of NHIS enrolment, renewal and drop out among older adults, defined as those aged 50 plus, to assess whether they differ from younger adults (aged 18-49). In addition, the paper is particularly interested in examining whether the correlates of insurance affiliation differ depending on which survey is used. The correlates of NHIS coverage among younger and older adults are assessed using both the GLSS and SAGE. Comparing different datasets to look for consistency in results, especially relating to the determinants of enrolment, adds to the evidence base for the NHIS.

Demand patterns of insurance enrolment will reveal what factors influence the decision to partake in the NHIS. Knowledge of who is enrolled in the NHIS and why others are not provides an understanding as to whether opportunities to enrol are equal or whether enrolment patterns vary depending on socioeconomic status, sex or residence. For progress to UHC to be achieved in times of ageing populations, health policies have to benefit all, inclusive of both younger as well as older adults [12]. The findings of this paper can be used by policymakers to implement target-orientated policy measures to increase enrolment in the NHIS.

### Enrolment in the National Health Insurance System in Ghana

In order to become a member of the NHIS, individuals first need to register at their local district office, pay a registration fee and a scaled-to-income premium. The health insurance membership ID card - which provides evidence of enrolment and entitles members to free use of care at accredited facilities - will then be provided for participants after a waiting period which can take up to several months [3]. Immediate biometric registration at selected district offices has been launched only recently to optimise the enrolment process [13]. The NHIS membership expires after one year, meaning that each year members require a renewal of insurance membership to remain eligible for NHIS benefits.

On reaching 70 years of age, individuals are exempt from the NHIS premium payment but not from the registration fee. In addition, individuals who receive a pension from the Social Security and National Insurance Trust (SSNIT) are exempt from the NHIS premium [14]. Other groups that are exempt from paying the premium include all children under 18 when both of their parents are enrolled in the NHIS, the ‘core poor’ (defined as those with no regular source of income and no fixed place of residence), and pregnant women.

## Methods

### Data

This paper uses two different datasets that aim to be nationally representative and include information on health insurance coverage in Ghana: the Ghanaian Living Standards Survey (GLSS) round 6 (2012/13) and the Study on Global Ageing and Adult Health (SAGE) wave 1 (2007/08).

Round 6 of GLSS interviewed 16,772 households (a response rate of over 93%) requesting information on all household members at all ages. The GLSS collected specific household level information on expenditure, income, assets and housing conditions as well as detailed information on household members’ socio-demographic characteristics, health, insurance status and employment.

The SAGE is a longitudinal study with the purpose of collecting rich and detailed data on older adults. The survey oversampled individuals that are aged 50 and over, while a smaller sample of individuals aged 18-49 years was collected as a control group [15]. A proxy questionnaire was completed for those individuals who were found to be unable to complete the questionnaire themselves due to cognition or health reasons. In the Ghanaian SAGE wave 1, only 17 people were proxy respondents. In total 5,110 individuals were interviewed during the first wave of the Ghanaian SAGE (response rate 80%).

### Measures

Reviewing the literature uncovers a substantial lack of studies that have examined the determinants of insurance coverage specifically among older adults in sub-Saharan Africa. Available studies have mainly focused on younger adults (for example [6, 16], or [17]). Existing studies informed the choice of key variables that were considered in the analysis.

Overall life expectancy in Sub-Saharan Africa is still low. Nine countries in the region still have an average life expectancy of less than 55 years [18]. Ghana has an average life expectancy of 62 years and the healthy life expectancy at birth is 54 years [19]. For the analysis in this paper, people aged 50 and above were classified as older adults. It is argued that the commonly used 60 or 65 cut-off point for defining an individual as an ‘older adult’ is not appropriate in the sub-Saharan African context.

The variable *age* has been transformed from a continuous into a categorical variable in order to control for changes in the demand for healthcare insurance among different age groups. Akazili, et al. [17] showed that reproductive age significantly affects NHIS enrolment and van der Wielen, et al. [20] found that age increases the likelihood of NHIS membership among older adults. Ten year age intervals were chosen for the analysis (18-29, 30-39, 40-49, 50-59, 60-69, 70+). These categories make theoretical sense, particularly for older adults, as they distinguish groups that may be differentially treated within the NHIS through regulations governing exemptions from payments. Sixty is the official retirement age in Ghana and all pensioners under the Ghanaian SSNIT Pension Scheme are exempt from premium payments. Those aged 60-69 years who do not receive pensions and work in the informal sector are, however, still liable for premium payments on the NHIS. It is anticipated therefore that due to the exemptions on premiums for people aged 70 plus, a sharp rise in insurance membership will be observed among those oldest-old adults.

Furthermore, education can increase awareness and understanding of the healthcare system and its advantages. Available literature shows that education positively influences the likelihood of being a member of the NHIS [7]. The variable *education* was recoded in a way that is sensitive to key milestones in the Ghanaian education system [21]. The variable was adjusted for changes in the school system to ensure that the school system in place when an older adult went to school was taken into account.

Previous studies reported that living standards determine insurance coverage in Ghana [5 16], with the poorest being least likely to be enrolled in the NHIS. Living standards are most commonly measured based on income, expenditure or consumption. Problems of misreporting and non-response of income components in surveys [22], as well as the lack of comparable expenditure data in the two surveys, make the use of proxy measures of living standards sensible. Information on household assets, which have been collected in both surveys, can be used as a proxy measure of prosperity [23]. Thus, this paper used consumer items (such as bicycle, television, and car) and dwelling characteristics (such as drinking water, type of flooring material, and sanitation facilities) to measure living standards. In order to take into account urban and rural differences in the distribution of assets, a wealth index was created over urban and rural residence using principal component analysis. Due to sample size issues, wealth tertiles were used.

Area characteristics are hypothesised to affect the demand for healthcare. *Place of residence* and *region* were included to capture regional differences. Here, it is hypothesised that living in an urban area increases the likelihood of health insurance enrolment due to the greater availablity of services. This is supported by Akazili, et al. [17] who found that compared to urban dewellers, rural residents are signifcanlty less likely to be covered by the NHIS.

Employment status was added to the analysis to account for premium exemptions for people who are contributing to the NHIS through automatic payroll reduction when working in the formal sector.

Other potential control variables were not directly comparable between the two surveys due to substantial differences in the questionnaire.

The GLSS asked about *former* insurance enrolment, hence only the GLSS was used for the analysis of insurance re-enrolment and drop out. One additional variable - disability status - was added to the model looking at re-enrolment and insurance drop out. Measuring health in terms of disabilities tries to minimise biases resulting from older adults’ tendency to overstate their health status in surveys as they have lower expectations of good health [24].

The coding of all control variables is summarised in Table 1.

**Table 1:** Description and coding of control variables

Variable	Coding
Sex	Coded dichotomously using the category male as reference.
Marital Status	Grouped into 4 categories: married/cohabiting; never married; separated/divorced and widowed. Reference category: married/cohabiting.
Education	Converted into 4 categories from none to tertiary education with no formal education as reference category.
Age	Treated as a categorical variable: *Younger adults:* (18-29,30-39,40-49) with 18-29 used as reference category *Older adults:* (50-59, 60-69, 70+) with 50-59 used as reference category.
Employment status	Converted into 2 categories with not currently employed in the informal sector as reference category.
Wealth	Wealth tertile. Reference category: Lower third.
Region	10 regions of Ghana. Reference: Greater Accra.
Residence	Coded dichotomously. Reference: Rural.
Disability	Coded dichotomously into yes -suffering from disabilities that limit his/her full participation in life activities- and no – not suffering from disabilities that limit his/her full participation in life activities. Latter was used as the reference category.

The characteristics of the study population using the SAGE and GLSS are summarised in Table 2. Table 2 highlights the differences between younger and older adults in the surveys used. It shows that in both surveys a higher proportion of older adults are living in rural areas compared with their younger counterparts, and that older adults are lower educated and suffer from higher levels of disablities.

**Table 2:** Characteristics of the study population

		SAGE				GLSS			
		18-49		50 plus		18-49		50 plus	
Variable	Measure	%	N	%	N	%	N	%	N
Sex	Male	48.89	437	52.43	2,202	46.03	13,129	45.73	4,337
	Female	51.11	352	47.57	2,014	53.97	15,187	54.27	5,167
Marital Status	Married	76.76	606	59.21	2,393	57.47	16,733	63.48	6,175
	Separated/Divorced	7.69	61	12.97	598	5.65	1,393	11.4	904
	Widowed	4.76	31	26.55	1,176	1.74	505	24.09	2,325
	Never married	10.79	91	1.27	49	35.15	9,685	1.03	100
Education	None	37.65	323	64.14	2,740	19.33	7,296	44.19	5,104
	Primary	21.74	165	11.04	459	56.83	15,095	43.63	3,462
	Secondary	35.91	269	21.28	869	17.51	4,312	6.23	457
	Tertiary	4.70	32	3.54	148	6.32	1,613	5.95	481
Age	Mean	37.81	789	64.30	4,216	31.01	28,316	62.62	9,504
Employment status	Employed in public sector	5.52	41	5.03	205	4.53	1,240	4.04	359
	Not employed in public sector	94.48	748	94.97	4,011	95.47	27,076	95.96	9,145
Wealth	Lower third	32.74	298	38.16	1,694	28.72	11,117	37.07	4,532
	Middle third	32.74	252	33.14	1,375	33.35	9,052	34.48	2,952
	Upper third	35.48	239	28.70	1,147	37.93	8,147	28.45	2,020
Region	Greater Accra	16.81	110	13.80	487	19.59	3,231	13.84	759
	Western	11.14	94	9.43	508	9.21	2,842	7.91	753
	Central	9.10	89	9.60	451	8.63	2,394	9.36	860
	Volta	8.20	73	9.64	400	8.05	2,495	9.37	984
	Eastern	10.07	75	12.28	546	9.77	2,635	12.96	1,131
	Ashanti	18.96	126	20.76	667	19.56	3,041	20.69	979
	Brong Ahafo	10.29	76	8.41	402	9.42	2,676	9.42	857
	Northern	7.94	67	8.36	381	9.33	3,567	8.45	1,010
	Upper East	4.79	45	4.45	251	3.81	2,569	4.89	1,043
	Upper West	2.69	34	3.26	123	2.64	2,866	3.13	1,128
Residence	Urban	48.00	329	41.00	1,724	54.82	12,067	46.90	3,391
	Rural	52.00	460	59.00	2,492	45.18	16,249	53.10	6,113
Disability	Yes	N/A		N/A		1.46	27,726	5.94	590
	No	N/A		N/A		98.54	434	94.06	8,863

*Source: SAGE 2007/2008, GLSS 2012/2013. Based on observations with complete information. All descriptive statistics are based on weights controlling for a multistage survey design.*

### Methods of Analysis

The initial analysis distinguishes between *NHIS coverage* and *NHIS non-coverage.* Bivariate analysis of the outcome and explanatory variables highlights patterns of NHIS enrolment, with Pearson’s Chi-squared test used to assess whether there is a significant association at a 5% significance level. Binary logistic regression was then applied to calculate the predicted probability of falling into the outcome category for different groups. Separate models were built for adults aged 18-49 and older adults aged 50 plus using the two different surveys.

Building upon this, supplementary analysis using the GLSS and a multinomial logistic regression model then distinguished between three outcomes of insurance status: *never enrolled in an insurance scheme*, *currently insured under the NHIS* (reference category) and *previously insured*. Out of the two surveys, only the GLSS asked about *previous* insurance enrolment, which made this supplementary analysis possible, however, findings could not be compared to the SAGE.

Cluster-robust standard errors are reported to account for the clustering of individuals within communities and households.

All analysis was carried out in STATA version 14 [25] and sample weights were applied throughout to adjust for unequal probability of selection.

## Results

### Determinants of Current Enrolment

**Fig. 1:**
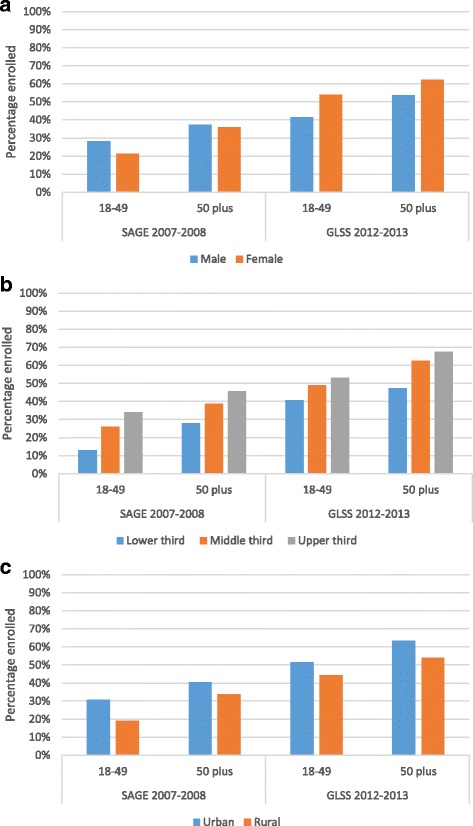
NHIS coverage by age group and (**a**) gender, (**b**) wealth and (**c**) region

In order to understand differences in current enrolment between younger and older adults, Fig.1 compares NHIS coverage between both groups and selected variables of interest. Overall, a higher enrolment rate is found among older adults compared to those aged 18-49 (*p* <0.001). Comparing NHIS coverage of 18-49 year olds by gender, the GLSS indicates a higher enrolment rate among females compared to males (see Fig. 1a). However, in contrast, the results from the SAGE data point to a higher level of coverage among males. Looking at older adults (aged 50 and over), both surveys reveal a higher enrolment rate among females.

Both surveys confirm that wealthier individuals are more likely to enrol in the NHIS (*p* <0.01) (Fig. 1b). This was found to be true for both age groups even though adults aged 70 plus are exempt from the premium. 47% of people aged 50 plus who belong to the poorest wealth tertile report NHIS coverage in the GLSS, compared to 67% in the richest tertile. The SAGE shows a similar differential by wealth status, with 28% of poorer older adults covered under the NHIS compared to 46% of rich older adults.

With regard to residential differences, both surveys show for both age groups a higher coverage in urban areas compared to rural areas (*p* <0.001) (Fig. 1c).

### Regression Results

Logistic regression analysis was conducted for each survey to determine the correlates of health insurance membership. Two models were fitted; one for adults aged 18-49, and one for older adults aged 50 plus. Results are summarised in Tables 3 and 4 and are presented separately by age group. Due to the different survey sample sizes, the presentation of the results focuses on the direction of associations rather than only on significance.

**Table 3:** Logistic regression results: Odds ratio of NHIS coverage among adults aged 18-49

		SAGE				GLSS			
		Odds Ratio		95% CI		Odds Ratio		95% CI	
Sex	Male	1.00				1.00			
	Female	0.80		0.50	1.29	1.99	***	1.86	2.13
Marital Status	Married	1.00				1.00			
	Separated/Divorced	1.10		0.52	2.33	0.80	*	0.69	0.93
	Widowed	1.12		0.42	3.02	1.07		0.82	1.39
	Never married	0.66		0.27	1.59	0.98		0.88	1.09
Education	None	1.00				1.00			
	Primary	2.01	*	1.12	3.61	1.45	***	1.29	1.62
	Secondary	1.29		0.70	2.37	2.45	***	2.13	2.83
	Tertiary	6.84	**	1.89	24.79	2.84	***	2.34	3.44
Age	18-29	1.00				1.00			
	30-39	1.19		0.57	2.47	1.03		0.94	1.13
	40-49	1.17		0.58	2.38	1.22	***	1.10	1.36
Employment status	Not employed in public sector	1.00				1.00			
	Employed in public sector	5.26	***	2.32	11.91	2.02	***	1.67	2.45
Wealth	Lower third	1.00				1.00			
	Middle third	3.61	**	1.53	8.50	1.47	***	1.29	1.68
	Upper third	4.65	**	1.89	11.41	1.71	***	1.44	2.02
Region	Greater Accra	1.00				1.00			
	Western	4.17	**	1.58	10.99	1.38		0.98	1.96
	Central	2.94	*	1.01	8.53	0.98		0.71	1.35
	Volta	2.19		0.65	7.38	1.71	**	1.26	2.32
	Eastern	4.45	**	1.69	11.71	1.92	***	1.39	2.66
	Ashanti	3.37	**	1.38	8.20	1.73	**	1.27	2.37
	Brong Ahafo	5.35	**	1.84	15.54	2.90	***	2.11	3.98
	Northern	9.66	***	2.89	32.27	1.83	**	1.29	2.60
	Upper East	11.97	**	2.47	58.02	6.81	***	4.65	9.97
	Upper West	47.19	***	13.40	166.19	9.02	***	6.06	13.43
Residence	Rural	1.00				1.00			
	Urban	1.51		0.87	2.61	1.14		0.97	1.32
*N*		*789*				*28,316*			

Source: SAGE 2007/2008, GLSS 2012/2013, *** p<0.001, ** p<0.01 * p<0.05

**Table 4:** Logistic regression results: Odds ratio of NHIS coverage among adults aged 50 plus

		SAGE				GLSS			
		Odds Ratio		95% CI		Odds Ratio		95% CI	
Sex	Male	1.00				1.00			
	Female	1.16		0.94	1.44	1.91	***	1.70	2.15
Marital Status	Married	1.00				1.00			
	Separated/Divorced	0.85		0.66	1.09	0.67	***	0.54	0.81
	Widowed	0.85		0.68	1.06	0.76	**	0.64	0.89
	Never married	0.50		0.24	1.06	0.36	***	0.21	0.64
Education	None	1.00				1.00			
	Primary	1.40	*	1.06	1.86	1.36	***	1.18	1.56
	Secondary	1.29		0.98	1.70	1.72	***	1.28	2.30
	Tertiary	2.99	***	1.75	5.12	2.43	***	1.70	3.46
Age	50-59	1.00				1.00			
	60-69	1.59	***	1.33	1.90	1.48	***	1.30	1.69
	70 plus	2.92	***	2.33	3.65	2.52	***	2.16	2.94
Employment status	Not employed in public sector	1.00				1.00			
	Employed in public sector	1.80	**	1.21	2.70	2.26	***	1.48	3.43
Wealth	Lower third	1.00				1.00			
	Middle third	1.65	***	1.28	2.13	1.93	***	1.63	2.29
	Upper third	2.72	***	2.03	3.64	2.86	***	2.23	3.67
Region	Greater Accra	1.00				1.00			
	Western	3.84	***	2.33	6.33	2.08	***	1.47	2.94
	Central	1.90	*	1.08	3.34	1.30		0.91	1.85
	Volta	2.79	***	1.71	4.53	2.53	***	1.81	3.52
	Eastern	6.31	***	3.88	10.25	2.92	***	2.10	4.07
	Ashanti	3.05	***	1.88	4.96	2.89	***	2.09	3.99
	Brong Ahafo	6.80	***	3.95	11.71	5.10	***	3.64	7.16
	Northern	3.41	***	1.78	6.52	1.91	**	1.30	2.80
	Upper East	3.27	***	1.70	6.29	6.02	***	3.84	9.45
	Upper West	6.42	***	3.15	13.09	7.62	***	4.92	11.82
Residence	Rural	1.00				1.00			
	Urban	1.16		0.88	1.54	1.25	*	1.04	1.49
*N*		*4,216*				*9,504*			

Source: SAGE 2007/2008, GLSS 2012/2013, *** p<0.001, ** p<0.01 * p<0.05

### Adults Aged 18 to 49

In line with the bi-variate results discussed above, Table 3 illustrates a difference in the effect of gender across the surveys. The GLSS highlights that females are significantly more likely to enrol in the NHIS (OR: 1.99 CI: 1.86-2.13). No significant association between gender and insurance coverage is found using SAGE, however, the direction of the effect suggests that women are less likely to enrol in the NHIS compared to men (OR: 0.80 CI: 0.50-1.29).

In the SAGE and the GLSS place of residence and marital status are found to have no significant effect on NHIS enrolment among younger adults. However, in both surveys higher levels of wealth are significantly and positively associated with insurance coverage. Similarly, higher education is associated with a greater likelihood of being enrolled in an insurance scheme. Further, those working in the public sector are more likely to be insured under the NHIS (SAGE OR: 5.26 CI: 2.32-11.91; GLSS OR: 2.02 CI: 1.67-2.45).

### Older Adults Aged 50 plus

Amongst people aged 50 plus, Table 4 shows an increasing likelihood of NHIS membership with age and wealth. Despite the premium exemption, richer older adults are still more likely to be part of the NHIS than poorer elders. In line with the findings for younger adults, those working in the formal public sector are significantly more likely to be insured under the NHIS (SAGE OR: 1.80 CI: 1.21-2.70; GLSS OR: 2.26 CI: 1.48-3.34).

Unlike for younger adults, marital status and place of residence are found to have a significant effect on insurance enrolment for older adults when using the GLSS. Non-married older adults are significantly less likely to enrol in the NHIS compared to their married counterparts. Table 4 shows a significantly greater likelihood of insurance enrolment in urban areas when using the GLSS (OR: 1.25 CI: 1.04-1.49). Looking at regional effects though, the two different surveys are consistent in the finding that residents of any other region were more likely to enrol in the NHIS than those living in Greater Accra.

When comparing both surveys, the analysis shows that the direction of association is consistent for most independent variables across both surveys.

**Fig. 2:**
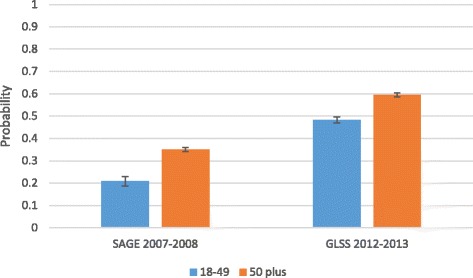
Probability of current NHIS coverage by age group and survey

As a final point, Fig. 2 highlights that older adults have a greater probability of being part of the NHIS compared to younger adults. Secondly, it highlights that among both population groups, the probability of being insured increases over time.

### Determinants of Previous Enrolment

Tables 5 and 6 present the odds ratio comparing the multiple factors that determine current, none, and previous insurance enrolment among adults aged 18-49 as well as adults aged 50 and over. The models give the odds ratio for either having never been enrolled or dropped out, in comparison with being currently enrolled.

**Table 5:** Multinomial logistic regression: Odds ratio of insurance status among adults aged 18-49

		Never enrolled				Prev. enrolled			
		Odds Ratio		95% CI		Odds Ratio		95% CI	
Sex	Male	1.00				1.00			
	Female	0.39	***	0.36	0.42	0.81	***	0.74	0.88
Marital Status	Married	1.00				1.00			
	Separated/Divorced	1.31	**	1.10	1.56	1.19		0.98	1.44
	Widowed	1.07		0.80	1.43	0.75		0.50	1.10
	Never married	0.98		0.87	1.11	1.11		0.97	1.28
Education	None	1.00				1.00			
	Primary	0.61	***	0.53	0.69	0.87		0.75	1.01
	Secondary	0.33	***	0.28	0.38	0.60	***	0.50	0.73
	Tertiary	0.25	***	0.19	0.31	0.63	**	0.49	0.82
Age	18-29	1.00				1.00			
	30-39	0.97		0.87	1.07	0.98		0.86	1.13
	40-49	0.77	***	0.68	0.86	0.94		0.80	1.11
Employment status	Not employed in public sector	1.00				1.00			
	Employed in public sector	0.38	***	0.30	0.48	0.69	**	0.54	0.87
Wealth	Lower third	1.00				1.00			
	Middle third	0.61	***	0.53	0.71	0.80	*	0.68	0.95
	Upper third	0.51	***	0.42	0.61	0.74	**	0.60	0.92
Region	Greater Accra	1.00				1.00			
	Western	0.50	***	0.35	0.73	1.46		0.99	2.17
	Central	0.95		0.67	1.34	0.91		0.60	1.37
	Volta	0.40	***	0.29	0.55	1.23		0.84	1.79
	Eastern	0.36	***	0.25	0.50	1.08		0.73	1.58
	Ashanti	0.33	***	0.24	0.45	1.58		1.09	2.28
	Brong Ahafo	0.17	***	0.12	0.24	1.15		0.81	1.64
	Northern	0.32	***	0.22	0.47	1.49	*	1.00	2.20
	Upper East	0.10	***	0.06	0.15	0.30	***	0.19	0.47
	Upper West	0.06	***	0.04	0.09	0.36	***	0.23	0.57
Residence	Rural	1.00				1.00			
	Urban	0.76	**	0.65	0.90	1.08		0.90	1.31
Disability	No	1.00				1.00			
	Yes	0.83		0.62	1.09	0.99		0.68	1.43
*N*		*28,160*				*28,160*			

Source: GLSS 2012/2013, *** p<0.001, ** p<0.01 * p<0.05

**Table 6:** Multinomial logistic regression: Odds ratio of insurance status among adults aged 50 plus

		Never enrolled				Prev. enrolled			
		Odds Ratio		95% CI		Odds Ratio		95% CI	
Sex	Male	1.00				1.00			
	Female	0.43	***	0.38	0.50	0.72	***	0.61	0.85
Marital Status	Married	1.00				1.00			
	Separated/Divorced	1.68	***	1.34	2.11	1.24		0.93	1.66
	Widowed	1.43	**	1.16	1.75	1.16		0.93	1.45
	Never married	2.65	**	1.48	4.74	2.17		1.00	4.70
Education	None	1.00				1.00			
	Primary	0.65	***	0.55	0.76	0.92	*	0.75	1.13
	Secondary	0.54	**	0.38	0.77	0.63	***	0.41	0.95
	Tertiary	0.37	***	0.24	0.57	0.49		0.30	0.79
Age	50-59	1.00				1.00			
	60-69	0.62	***	0.53	0.73	0.80	*	0.66	0.97
	70 plus	0.33	***	0.28	0.39	0.57	***	0.45	0.71
Employment status	Not employed in public sector	1.00				1.00			
	Employed in public sector	0.22	***	0.13	0.37	0.98		0.57	1.67
Wealth	Lower third	1.00				1.00			
	Middle third	0.48	***	0.39	0.58	0.60	***	0.47	0.76
	Upper third	0.31	***	0.23	0.41	0.43	***	0.32	0.58
Region	Greater Accra	1.00				1.00			
	Western	0.34	***	0.24	0.50	0.94		0.57	1.52
	Central	0.72		0.49	1.05	0.78		0.48	1.26
	Volta	0.26	***	0.18	0.37	0.88		0.56	1.38
	Eastern	0.24	***	0.17	0.34	0.67		0.42	1.09
	Ashanti	0.22	***	0.16	0.31	0.82		0.52	1.28
	Brong Ahafo	0.09	***	0.06	0.14	0.63	*	0.40	0.98
	Northern	0.35	***	0.23	0.53	1.12		0.68	1.84
	Upper East	0.11	***	0.07	0.18	0.37	***	0.21	0.67
	Upper West	0.07	***	0.05	0.12	0.37	***	0.20	0.69
Residence	Rural	1.00				1.00			
	Urban	0.70	***	0.57	0.86	1.01		0.79	1.30
Disability	No	1.00				1.00			
	Yes	0.89		0.66	1.18	0.90		0.65	1.25
*N*		*9,453*				*9,453*			

Source: GLSS 2012/2013, *** p<0.001, ** p<0.01 * p<0.05

**Fig. 3:**
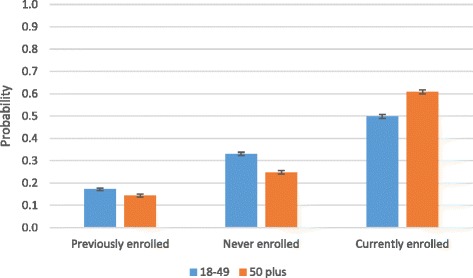
Predicted probability of insurance status by age group

Figure 3 shows the predicted probability of insurance status by age group. It shows that older adults have a slightly lower probability of dropping out of insurance coverage (14%) compared to younger adults (17%).

**Fig. 4:**
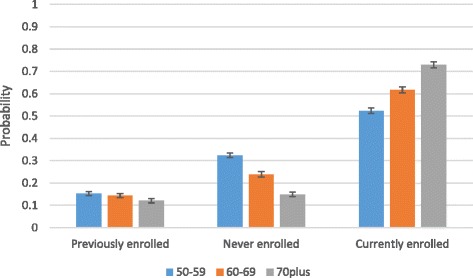
Predicted probabilities of insurance status among older adults

Due to the premium exemption from age 70, it is of interest to unpack those findings further for older adults. Figure 4 shows that older adults aged 70 plus have a significantly smaller likelihood of dropping out of insurance coverage and are more likely to be currently enrolled compared to other older adults aged between 50 and 69.

Further results once more confirm that insurance status is strongly related with wealth and educational status. For both younger and older adults the probability of dropping out of insurance enrolment is significantly higher for the poorest and lower educated. Moreover, the likelihood of dropping out of insurance enrolment decreases significantly with being female for both age groups. Separating the analysis between never, currently, and previously enrolled shows that younger and older adults living in rural areas are significantly more likely to have never been enrolled in an insurance scheme.

## Discussion

The lack of social security and increasing healthcare demands in older age suggest that the establishment of comprehensive universal health insurance systems may be particularly valuable in ensuring equal access to healthcare in sub-Saharan Africa. The study carried out here advances the literature on insurance enrolment by analysing factors associated with insurance enrolment among older adults in Ghana as a separate population group. It indicates that correlates of insurance membership overlap between younger and older adults with only minor exceptions. This suggests that healthcare reforms to increase insurance affiliation can be beneficial across both population groups. Such a finding appears especially clear when it comes to promoting higher coverage among the poor. Despite scaled-to-income fees that, in theory, should mitigate skewed enrolment according to wealth, the findings suggest that insurance enrolment increases with wealth. This finding confirms that of other researchers e.g. [7, 16, 26]. Despite the theoretical argument that health insurance will improve access to healthcare, low enrolment among the poorest indicates a failure of policy to ensure equal access. For example, local district offices in Ghana have been found to be charging flat premium payments across the board due to difficulties in determining the socioeconomic group [27]. More research is needed on whether the "core poor" and people over 70 are aware of the premium exemption. Awareness of social protection schemes seems to be a problem in Ghana. Other social protection programmes, including the Livelihood Empowerment Against Poverty (LEAP), have shown low awareness and a low uptake [28]. More research is needed on whether the registration fee hinders enrolment as, even for those exempt from the insurance premium itself, the registration fee is a requirement. Kusi and Enemark [29] revealed that the registration fee imposes a burden on very poor households.

In order to overcome the gap in insurance enrolment between rich and poor, external financial support needs to be increased and premiums need to decrease. Government subsidies or donor funding are seen as one path to stabilizing the health finance system in the long run. To increase enrolment, efforts need to be undertaken to ensure that all people who fall below the poverty line and are aged 70 and over are aware of the premium exemption in the NHIS. To improve insurance coverage among the poor, additional policy initiatives should be considered such as travel reimbursement, particularly for those living in rural areas where insurance coverage was found to be low.

An increase in education was found to significantly increase insurance affiliation for both younger and older adults. This finding underlines that of Ayitey, et al. [7] who showed that with rising education, NHIS enrolment increases. It is unlikely that there is a direct causal link between higher education and increased NHIS awareness among older adults. In the GLSS round 6 (2012/2013) less than 0.5% of respondents reported that they did not know about the system. Also Schultz, et al. [30] found no evidence that health education increased NHIS enrolment. Further research is needed to fully understand and explain the differences in NHIS enrolment by education status.

Comparing NHIS coverage of 18-49 year olds by gender, the GLSS shows a higher enrolment rate among females compared to males. This is in line with previous research by Ayitey, et al. [7] who also found that the NHIS uptake in Ghana is greater among women compared to men. However, in contrast, the results from the SAGE data point to a higher level of coverage among males. These differences can, in part, be attributed to differences in the timing of the fieldwork and the policy environment. Since July 2008, all pregnant women who attend antenatal care in one of the NHIA accredited facilities are exempt from the NHIS premium payment as they are automatically registered in the scheme upon arrival. They and their newborn child are entitled to NHIS benefits until 3 months after the delivery [31]. The fieldwork for the SAGE took place between May 2007 and June 2008. This means that during the SAGE data collection period, the premium exemption for pregnant women was not yet in place, which could account for the lower coverage among women particularly in reproductive age. Both surveys showed significantly lower likelihood of insurance drop out among females in both age groups. Women might be more aware of the benefits provided by the NHIS as women tend to be responsible for the health and wellbeing of the household [6].

With regards to age, this study found that an increase in age among older adults is associated with a greater probability of being insured in the NHIS. This refers back to the hypothesis that people assess their health status before insurance enrolment [32] and that older adults are more likely to invest in their health due to increasing usage of healthcare with age. Further analysis in this paper showed that older adults also had a slightly lower probability of dropping out of insurance coverage compared to younger adults. This could be due to the above-described greater need of health insurance coverage with increasing age. In addition, older adults might have had more time to re-enrol after dropping out. The opportunity cost of going to the district office to enrol tends to be lower for older adults as they are less likely to have to take a day off work. Younger adults tend to have less time to enrol and have greater opportunity costs like missing time at work. Following this argument, older adults may also have had more opportunities to enrol in the first instance. Younger adults may have dropped out but not yet re-enrolled as they need less healthcare than older adults. Women especially might not feel the need for insurance coverage after childbirth.

When treating the surveys as two cross-sectional surveys, they indicate an increasing trend in NHIS enrolment. Trends in enrolment can be attributed to changes in health polices and economic circumstances in Ghana including NHIS facility accreditation in 2009 to improve quality and access to care, free enrolment for pregnant women in 2008, economic growth and increasing per capita income, and more general improvements of the NHIS over time. In 2007/2008, when the SAGE was conducted, the NHIS was still a new system. Over 10 years on from its implementation, it has now been allowed some time to learn from experience and improve its services.

Finally, this paper was interested in answering the question of whether the correlates of insurance affiliation differ depending on which survey (GLSS or SAGE) is used. Because of the fact that the true population correlates of insurance coverage are unknown, it is difficult to estimate which of the surveys delivers more accurate results. Comparing survey results can,  however, give reassurance to analyses of the determinants of NHIS coverage. The findings give assurance that both datasets (GLSS and SAGE) are suitable for research on insurance affiliation and that both surveys produce comparable results.

## Conclusion

Using both timely and also less recent surveys indicates that although the gap in coverage among rich and poor and urban and rural residents appears to have decreased, these factors still determine NHIS coverage of younger and older adults. The same holds for education. Increased efforts are needed to ensure equal access to care in moving towards UHC. The results in this paper highlight the need to introduce specific measures to target those groups with low enrolment and those who are at the highest risk of dropping out. The existence of insurance alone is not sufficient and efforts are needed to target the poorest and less well educated members of society at all ages to ensure equal access to care across the life course.

## Abbreviations

DALYs: Disability-adjusted life-years
GLSS: Ghana Living Standards Survey
NHIA: National Health Insurance Authority
NHIS: National Health Insurance Scheme
SAGE: Study on Global Ageing and Adult Health
SSNIT: Social Security and National Insurance Trust
UHC: Universal Health Coverage
WHO: World Health Organization
